# Mechanisms of long non-coding RNAs in biological phenotypes and ferroptosis of glioma

**DOI:** 10.3389/fonc.2022.941327

**Published:** 2022-07-14

**Authors:** Xianyong Yin, Jiajia Gao, Zihao Liu, Min Han, Xiaoshuai Ji, Zhihai Wang, Yuming Li, Dong He, Fenglin Zhang, Qian Liu, Tao Xin

**Affiliations:** ^1^ Department of Neurosurgery, Shandong Medicine and Health Key Laboratory of Neurosurgery, The First Affiliated Hospital of Shandong First Medical University and Shandong Provincial Qianfoshan Hospital, Jinan, China; ^2^ Department of Neurosurgery, Shandong Provincial Qianfoshan Hospital, Cheeloo College of Medicine, Shandong University, Jinan, China; ^3^ Department of Neurosurgery, Shandong Provincial Hospital, Cheeloo College of Medicine, Shandong University, Jinan, China; ^4^ Department of Histology and Embryology, Cheeloo College of Medicine, Shandong University, Jinan, China; ^5^ Department of Neurosurgery, Jiangxi Provincial People’s Hospital Affiliated to Nanchang University, Nanchang, China; ^6^ Medical Science and Technology Innovation Center, Shandong First Medical University and Shandong Academy of Medical Sciences, Jinan, China

**Keywords:** lncRNAs, glioma, phenotypes, ferroptosis, mechanism

## Abstract

Glioma, one of the most common malignant tumors in the nervous system, is characterized by limited treatment, high mortality and poor prognosis. Numerous studies have shown that lncRNAs play an important role in the onset and progression of glioma by acting on various classical signaling pathways of tumors through signaling, trapping, guiding, scaffolding and other functions. LncRNAs contribute to the malignant progression of glioma *via* proliferation, apoptosis, epithelial-mesenchymal transformation, chemotherapy resistance, ferroptosis and other biological traits. In this paper, relevant lncRNA signaling pathways involved in glioma progression were systematically evaluated, with emphasis placed on the specific molecular mechanism of lncRNAs in the process of ferroptosis, in order to provide a theoretical basis for the application of lncRNAs in the anticancer treatment of glioma.

## Introduction

Human glioma, histologically originates from the neuroectoderm and is recognized as the most prevalent and lethal intracranial tumor, accounting for more than 50% of cerebral tumors (1, 2). Based on the malignancy characteristics, glioma can be classified as WHO grade I-IV. In addition, the latest version of the WHO classification divides glioma into more biologically and molecularly defined pathological subtypes (3). Currently, glioma is still difficult to treat. Although the current therapeutic schemes have advanced in operation, radiotherapy and chemotherapy, the prognosis of glioma patients still remains pessimistic due to the high rates of relapse and inevitable metastasis (4). Thus, it is critical to determine the exact molecular mechanisms leading to glioma onset and progression.

Long non-coding RNAs (lncRNAs) are the molecules that are more than 200 nucleotides in length and have no/little protein-coding functions/potentials and/or lack open reading frames. LncRNAs can modulate gene expression at the transcriptional or post-transcriptional levels (5), and several lines of evidence have shown that lncRNAs have been associated with the occurence and development of many human tumors, including glioma. More specifically, lncRNAs are involved in modulating the development of malignant glioma cells by altering cellular proliferation, apoptosis, drug resistance and ferroptosis (6). LncRNAs have been associated with the onset and development of many human malignant tumors, including glioma.

Ferroptosis is a cell death pathway characterized by iron dependency and excessive lipid peroxidation, making it unique in comparison to other cell death process such as apoptosis, necrosis and pyroptosis. According to the recent 2018 consensus derived from the nomenclature committee on cell death, ferroptotic cell death is a type of regulated cell death (RCD), in contrast to accidental cell death (ACD) which is caused by physical, chemical or other factors (7). Ferroptosis is a double-edged sword that plays a dual role in tumors *via* damage-associated molecular patterns (DAMPs) (8, 9). In this review, we summarized the functions and mechanisms of lncRNAs in the genesis and malignant development of glioma. We conclude that lncRNAs play a crucial role in glioma ferroptosis, contributing to deeply understand glioma pathogenesis and provide future directions for others.

## Classification and molecular mechanism of lncRNAs

**Figure 1:**
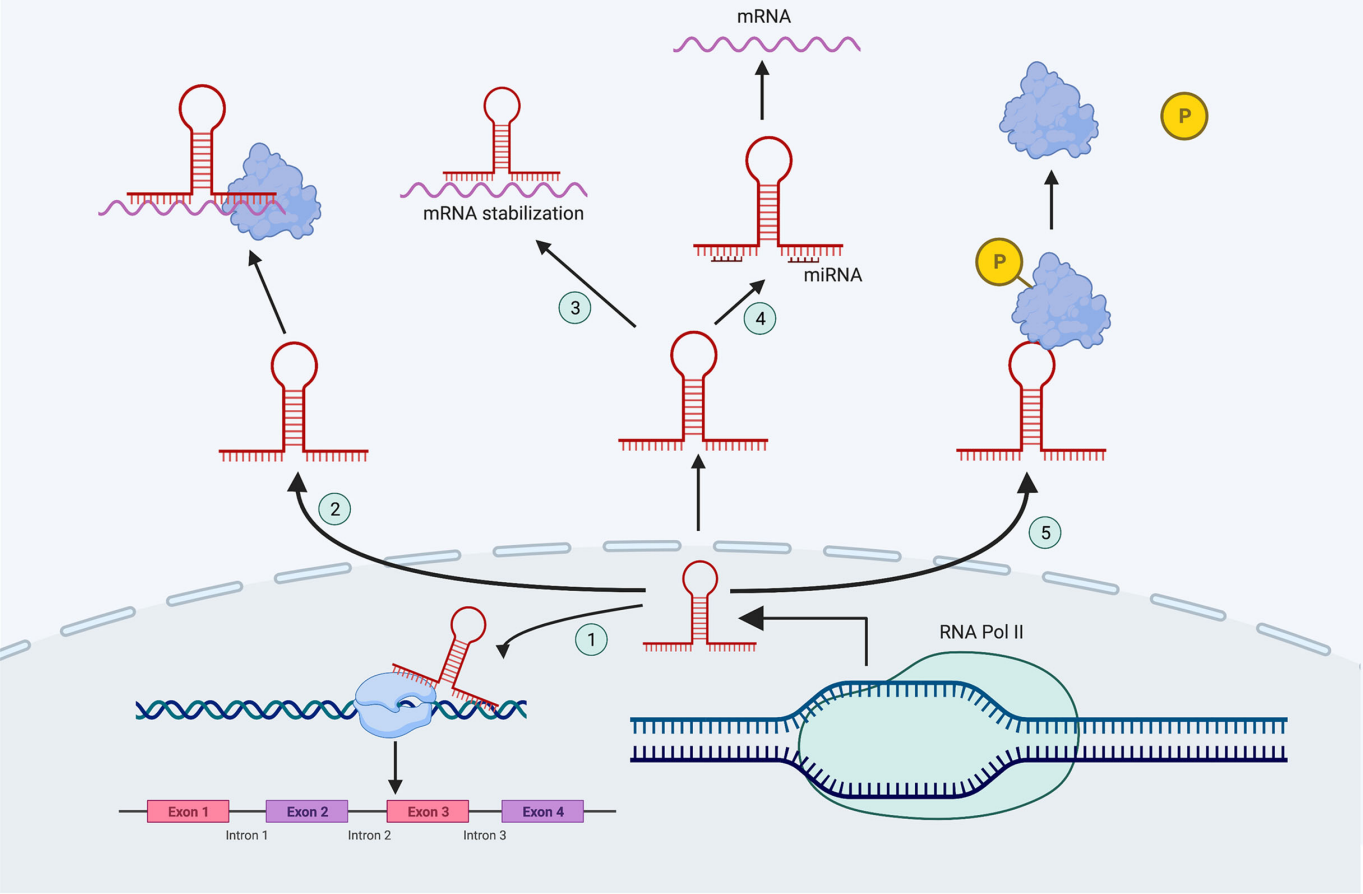
The mechanisms of lncRNAs. ① Molecular guide; ② Scaffolds; ③ Signals; ④ CeRNAs; ⑤ Molecular decoys.

According to their position in the reference genome, lncRNAs can be classified into the following five categories: sense, antisense, bidirectional, intronic and intergenic (10). According to their mechanism of action, lncRNAs can be divided into five functional types, and each lncRNA can coexist with multiple functions (**Figure 1**). 1) Molecular guide. LncRNAs act as molecular guides of ribonucleoprotein complexes to specific sites on chromatin (11). 2) Scaffolds. LncRNAs bind different effector molecules as binding scaffolders of protein complexes, and combine with these effector proteins to jointly regulate gene transcription in time and space (12). 3) Signals. LncRNAs stimulate a variety of signaling molecules to regulate their downstream signaling pathways during cell transcription (13). 4) Competitive endogenous RNAs (ceRNAs).

LncRNAs can be used as miRNA sponges that contain miRNA binding sites where miRNAs are sequestered, inhibiting miRNA target genes (14). 5) Molecular decoys. LncRNAs can act as molecular decoys *via* allosteric binding to specific proteins to inhibiting the function of downstream proteins (15).

## LncRNAs regulate the malignant progression of glioma

Abnormal expression of lncRNAs in glioma has been revealed to be interrelated tightly with the prognosis of glioma patients including overall survival (OS) and survival quality. For instance, lncRNA ROR1-AS1 was up-regulated in glioma and indicated a poor clinical outcome. Kaplan–Meier curves indicated that the 5-year survival rate of glioma patients was obviously higher in patients with lower ROR1-AS1 expression (16). Moreover, LINC01494 was over-expressed in glioma and was associated with a poor prognosis in glioma patients (17).Chen et al. analyzed the relationship between the expression of lncRNA CPS1-IT1 and the pathological characteristics of glioma, and found that low expression of lncRNA CPS1-IT1 led to elevated WHO grade and poor prognosis (18). In addition, the expression of lncRNA CASC7 was related to glioma progression and WHO stage, and was positively correlated with patient prognosis (19).

### LncRNAs and the proliferation of glioma

As one of the most important biological characteristics of tumor cells, cellular proliferation determines the occurrence and development of tumors. Previous studies have shown that lncRNAs ultimately affect the proliferation of glioma cells through a series of downstream pathways (**Table 1**).

**Table 1:** Representative lncRNAs and related signaling pathways in glioma proliferation.

LncRNA	Expression	Downstream Targets	Proliferation	References
SNHG20	upregulated	P21, CCNA1	promote	(20)
SNHG3	upregulated	EZH2, KLF2, P21	promote	(21)
SNHG6	upregulated	P21	promote	(22)
SNHG16	upregulated	P21, caspase 3/9, cyclinD1/B1	promote	(23)
RP11-732M18.3	upregulated	14-3-3β/α, UBE2E, P21	promote	(24)
ADAMTS9-AS1	upregulated	Wnt/β-catenin pathway	promote	(25)
H19	upregulated	miR-342, Wnt5a/β-catenin pathway	promote	(26)
CTBP1-AS2	upregulated	miR-370-3p, Wnt7a/β-catenin pathway	promote	(27)
SNHG20	upregulated	PTEN/PI3K/AKT pathway	promote	(28)
XIST	upregulated	miR-126, IRS1/PI3K/Akt pathway	promote	(29)
LBX2-AS1	upregulated	PI3K-Akt-GSK3β pathway	promote	(30)

P21, as a cyclin-dependent kinase inhibitor encoded by the CDKN1A gene, inhibits the formation of the CDK2-CDK1 complex and mediates the G1 phase arrest of p53- dependent cell cycle. Numerous studies have shown that lncRNAs regulated the cell cycle by affecting the expression of p21 protein, leading to the proliferation of glioma cells. For example, a study (20) comparing 108 glioma to control tissue samples found that lncRNA SNHG20 was highly expressed in glioma tissue and negatively correlated with patient prognosis. Further study of the specific mechanism showed that lncRNA SNHG20 accelerated the G0/G1 cycle by reducing p21 transcription, ultimately leading to the glioma cell proliferation. LncRNA SNHG3 was also highly expressed in glioma tissues and promoted the proliferation of glioma cells. It recruited EZH2 to the promoters of KLF2 and p21, and epigenetically inhibited KLF2 and p21 (21).

Moreover, lncRNA SNHG6 and SNHG16 promoted the proliferation of glioma cells *via* reducing p21 mRNA levels (22, 23). In addition to small nucleolar RNA host gene (SNHG), lncRNA RP11-732M18.3 induced the degradation of p21 and increased the proliferation of glioma cells. It recruited 14-3-3β/α to UBE2E1, and the binding of 14-3-3β/α to UBE2E1 enhanced the degradation activity of UBE2E1 on p21 *via* ubiquitination (24).

Another important molecular pathway activated during the development of human cancers, including glioma, is the Wnt/β-catenin signaling pathway (31, 32). LncRNA ADAMTS9-AS1 was confirmed to be involved in the positive regulation of Wnt/β-catenin signaling pathway in glioma, leading to glioma cell proliferation (25). Zhou et al. showed that lncRNA H19, as a ceRNA, directly bound to miR-342 and inhibited its expression. Knockdown of miR-342 in turn promoted Wnt5a and β-catenin expression to positively regulated the Wnt5a/β-catenin signaling axis and glioma cell proliferation (26). Similarly, lncRNA CTBP1-AS2 also functioned as a ceRNA and specifically bound miR-370-3p to inhibit its expression. Sequestration of miR-370-3p by CTBP1-AS2 prevented miR-370-3p 3’UTR binding and disinhibition of Wnt7a, and miR-370-3p knockdown activated Wnt7a/β-catenin signaling. Both actions accelerated the proliferation of glioma cells (27).

Several investigations have demonstrated that inhibition of the PI3K/AKT signaling pathway blocked cellular proliferation and played an anti-tumor role by inhibiting the cell cycle and inducing apoptosis (33, 34). For instance, lncRNA SNHG20 promoted the activation of the PI3K/AKT signaling pathway and accelerated the proliferation of glioma cells by inhibiting PTEN (28). LncRNA XIST, a molecular sponge of miR-126, promoted glucose metabolism and led to the glioma cell proliferation through regulation of the IRS1/PI3K/Akt pathway (29). LncRNA LBX2-AS1 knockdown caused a significant decrease in both GSK3β and Akt phosphorylation, suggesting that it promoted cell proliferation by activating the PI3K-Akt-GSK3β pathway (30).

### LncRNAs and apoptosis in glioma

Apoptosis, or programmed cell death, is strictly regulated at the genetic level, resulting in the orderly and efficient elimination of damaged cells (35). As an important biological process of cell metabolism, apoptosis is affected by many factors and is involved in the activation, expression and regulation of a series of genes. Dysfunctional apoptosis is closely related to tumorigenesis. At present, lncRNAs have been confirmed to activate or inhibit the apoptosis of glioma cells through downstream molecules (**Table 2**).

**Table 2:** Representative lncRNAs and related signaling pathways in glioma apoptosis.

LncRNA	Expression	Downstream Targets	Apoptosis	References
FOXD2-AS1	upregulated	EZH2, P53 pathway	inhibit	(36)
SNHG20	upregulated	miR-4486, MDM2-P53 pathway	inhibit	(37)
ST7-AS1	downregulated	PTBP1, Wnt/β-catenin pathway	promote	(38)
ANCR	upregulated	PTEN, EZH2, Bax, Bcl-2	inhibit	(39)
LOC101928963	upregulated	PMAIP1	inhibit	(40)
GAS5	downregulated	GSTM3, Caspase 3/7	promote	(41)
PCED1B-AS1	upregulated	miR-19-5p, PCED1B	inhibit	(42)

Traditionally, p53-induced apoptosis was considered a main mechanism that inhibited tumor development by regulating downstream target genes. At present, numerous P53 target genes are involved in apoptosis regulation, which can be mainly divided into two categories: death receptor family and the bcl-2 family. Numerous studies have shown that lncRNAs regulated the expression and degradation of p53 through a variety of downstream molecules, thus affecting the glioma cell apoptosis. LncRNA FOXD2-AS1 was significantly upregulated in glioma tissues and mainly distributed in the nucleus. By binding to EZH2, FOXD2-AS1 weakened the recruitment ability of p53, thus inhibiting glioma cell apoptosis and promoting malignant progression of glioma (36). LncRNA SNHG20 increased MDM2 level by binding miR-4486, which enhanced the degradation of P53 protein and ultimately inhibited the apoptosis of glioma cells (37). In addition, studies have confirmed that p53 binds to the lncRNA ST7-AS1 promoter to increase its transcription. Subsequently, lncRNA ST7-AS1 regulated p53 expression by binding to PTBP1, and forming a positive feedback loop to inhibit the progression of invasive glioma (38).

Apoptosis is regulated by many genes, among which the bcl-2 and caspase families are the most important. Bcl-2 and bax genes are important regulatory apoptotic genes that act antagonistically to each other in apoptosis regulation, and caspase-3 is a critical apoptotic execution protease. Numerous studies have proved that lncRNAs regulate the apoptosis of glioma cells by acting on them. Specifically, LncRNA ANCR regulated PTEN expression *via* binding and interacting with EZH2, thus inhibiting the apoptosis of glioma cells. Moreover, high expression of lncRNA ANCR reduced bax expression and promoted bcl-2 expression to produce an anti-apoptotic effect (39). Similarly, lncRNA LOC101928963 inhibited PMAIP1 expression, which also induced bcl-2 and reduced bax expression, and ultimately inhibited the apoptosis of glioma (40). Furthermore, lncRNA GAS5 increased Caspase-3/7 activity and promoted apoptosis *via* regulating GSTM3 (41). LncRNA PCED1B-AS1, on the other hand, inhibited caspase-3 activity *via* miR-19-5p/PCED1B axis, thereby activating glioma proliferation and limiting apoptosis (42).

A large amount of evidence has confirmed the strong correlation between P53 and lncRNAs, and these lncRNAs regulate tumor apoptosis as regulatory factors or effectors of P53. In addition, Liu et al. reported multiple lncRNAs expression levels under various antitumor drugs. By detecting the expression changes of lncRNAs in doxorubicin and resveratrol treated glioma cells, MIR155HG was up-regulated in response to resveratrol-induced apoptosis, GAS5 was up-regulated during doxorubicin-induced apoptosis, and MEG3 and ST7OT1 were up-regulated under apoptosis induced by both agents (43). These results indicate that lncRNAs can be used as targets of multiple chemotherapy drugs to promote glioma cell apoptosis, and a more complete lncRNAs action network is conducive to the development of more therapeutic targets and new chemotherapy drugs.

### LncRNAs and the EMT process in glioma

Epithelial-mesenchymal transformation (EMT) refers to the transformation of epithelial-to-mesenchymal cells and is recognized as an integral part of glioma invasion and migration. EMT is characterized by the loss of cell adhesion, changes in cytoskeletal components and the acquisition of migration and invasion characteristics (44). In addition to the invasion process, apoptosis, chemotherapy and immunotherapy resistance during glioma progression are also involved in EMT (45). EMT regulation is a complex network that includes multiple signaling pathways involving the TGFβ family, Wnts, Notch, EGF, HGF, FGF and HIF. Numerous studies have confirmed that lncRNAs regulated the EMT process of glioma cells through downstream pathways (**Table 3**).

**Table 3:** Representative lncRNAs and related signaling pathways of EMT process in glioma.

LncRNA	Expression	Downstream Targets	EMT	References
linc00645	upregulated	miR-205-3p, ZEB1	promote	(46)
UCA1	upregulated	miR-204-5p, ZEB1	promote	(47)
HOTTIP	upregulated	miR-101, ZEB1	promote	(48)
HOXC-AS2	upregulated	miR-876-5p, ZEB1	promote	(49)
CTBP1-AS2	upregulated	miR-370-3p, Wnt7a/β-catenin pathway	promote	(27)
H19	upregulated	Vimentin, ZEB1, Wnt/β-Catenin pathway	promote	(50)

Zinc-finger E-box-binding homeobox 1 (ZEB1) is an important regulator of EMT. LncRNA was known to function as a ceRNA to regulate ZEB1 *via* multiple pathways in regulation of EMT process of glioma cells (46). LncRNA linc00645, for instance, played a key role in TGF-β-triggered glioma cell EMT through competing with miR-205-3p and promoting the expression of downstream molecule ZEB1 (47). LncRNA UCA1 partially rescued the inhibitory effect of miR-204-5p on ZEB1 *via* binding and inhibiting miR-204-5p, which promoted the EMT process of glioma cells (48). Hypoxia-induced glioma cells upregulated lncRNA HOTTIP and sponge inhaled endogenous miR-101, resulting in increased ZEB1 expression and promoting EMT process (49). LncRNA HOXC-AS2 formed a positive feedback loop with ZEB1 through miR-876-5p to regulate the EMT in glioma, providing a potential therapeutic target for glioma prevention (50).

The Wnt signaling pathway also plays an important biological role in EMT in glioma. In this sense, lncRNA CTBP1-AS2 regulated the Wnt7a-mediated EMT by binding miR-370-3p (27), whereas lncRNA H19 inhibited EMT Wnt/β -catenin pathway (51).

In conclusion, a variety of lncRNAs can regulate the EMT process of glioma through ZEB1, which is closely related to tumor metastasis and drug resistance. ZEB1, a zinc-finger transcription factor induces EMT by regulating E-cadherin and vimentin. In-depth understanding of the molecular mechanism of lncRNAs control of EMT can not only reveal the process of metastatic drug resistance of tumor cells, but also provide new therapeutic targets and treatment options for effective cancer treatment.

### LncRNAs and TMZ resistance in glioma

Chemotherapy is a common postoperative treatment strategy for glioma treatment (52). Temozolomide (TMZ) is a second-generation oral alkylating agent that can easily cross the blood-brain barrier, therefore it is the standard first-line chemotherapy agent in the clinical treatment of glioma (53, 54). TMZ exerts its antitumor effects mainly through inducing base mismatch, DNA repair aberration, DNA chain break and cell death (55). However, TMZ can only slightly improve the survival of patients with glioma, because many patients develop resistance to TMZ, resulting in poor or no response to it (56).

At present, a string of studies have described the mechanism of glioma drug resistance to chemotherapy, and these mechanisms may involve lncRNAs (**Table 4**) and the β-catenin signaling pathway. LncRNA RMRP modulated TMZ resistance in glioma by regulating ZNRF3 levels and the Wnt/β-catenin signaling pathway to form a positive feedback loop (57). LncRNA MIR155HG was highly expressed in glioma tissues and promoted glioma resistance to TMZ by binding PTBP1 to regulate the Wnt/β-catenin pathway (58). It was found that lncRNA SOX2OT reduced the methylation level of SOX2 by interacting with ALKBH5, thus improving the SOX2 expression and activating the Wnt5a/β-catenin signaling pathway to promote TMZ resistance in glioma cells (59). In addition, lncRNA SNHG15 also activated the β-catenin signaling pathway by promoting SOX2 expression (60).

**Table 4:** Representative lncRNAs and related signaling pathways of TMZ resistance.

LncRNA	Expression	Downstream Targets	TMZ resistance	References
RMRP	upregulated	ZNRF3, Wnt/β-catenin pathway	promote	(56)
MIR155HG	upregulated	PTBP1, Wnt/β-catenin pathway	promote	(57)
SOX2OT	upregulated	SOX2, Wnt5a/β-catenin pathway	promote	(58)
SNHG15	upregulated	miR-627-5p, CDK6, SOX-2	promote	(59)
SNHG12	upregulated	miR-129-5p, MAPK1, E2F7	promote	(60)

TMZ resistance in glioma cells may be epigenetically regulated by lncRNAs. For example, one report showed that lncRNA SNHG12 was activated by DNA methylation in the promoter region CpG island, and lncRNA SNHG12 regulated the MAPK/ERK signaling pathway and G1/S cell cycle transition through competitive binding of miR-129-5p. Thus, DNA methylation of lncRNA SNHG12 ultimately regulated TMZ resistance in glioma cells (61).

Postoperative temozolomide chemotherapy has become the standard treatment for glioma. However, acquired TMZ resistance limits the treatment of patients with glioma, especially relapsing glioma. As mentioned above, some lncRNAs are associated with glioma drug resistance, which involves not only intracellular processes but also factors in the glioma microenvironment. Elucidate the molecular mechanism of TMZ resistance, which is helpful to rationally design the combined treatment plan to block TMZ chemotherapy resistance.

## LncRNAs and ferroptosis in glioma

As a matter of fact, iron is an essential nutrient and microelement for cell growth, no exception for cancer cells. Moreover, iron-dependent ferroptosis induces inflammation reaction to promote the initiation and advancement of cancers in early stages. On the other hand, cancer can be restrained by anti-cancer immune response triggered by ferroptosis and the release of damage-associated molecular pattern (DAMPs). Up to now, lncRNAs owing to diversities and complex functions is thought to be closely related to ferroptosis of various diseases based on explosive growing studies. Zhang et al. found that (62) curcumenol could hinder the progression of lung cancer by slowing down the multiplication and accelerating cell death as an effectual component of Wenyujin. Finally, they verified that lncRNA H19 could enhance the transcription activity of ferritin heavy chain1 (FTH1), a biomarker of ferroptosis, by interacting with miR-19b-3p as a competent endogenous RNA. Shi et al. found that (63) lncRNA AAB expressed highly and increased Fe^2+^ level to exert antitumor effect in cardiac microvascular endothelial cells (CMECs). Furthermore, they demonstrated that lncRNA AAB caused the disturbance between MMP9 and TIMP1 balance by sponging miR-30b-5p in CMECs. They even constructed a nanocomplex delivering si-lncRNA AAB into CMECs to provide a potential treatment method for cardiac hypertrophy patients. Besides, Luo et al. found that (64) lncRNA RP11-89 heightened the migration and expansion of bladder cancer *via* the miR-129-5p/PROM2 axis. It is acknowledged that prominin2 (PROM2) is the key molecule to inhibit ferroptosis. Evidence showed PROM2 executed a crucial role in the traffic of iron mediated by transferrin and altered the sensitive of cancer cells to ferroptosis.

### The metabolism of iron

Iron is one of the most indispensable metals for humans. Biological iron participates in various metabolic processes, including cellular proliferation and death, especially ferroptotic cell death. Intracellular iron exists in two oxidative states, Fe^2+^ and Fe^3+^, which can be randomly converted into different forms. Iron can be transported by binding to serum transferrin (TF) or lactotransferrin as Fe^3+^. Endocytosis occurs when serum TF binds to transferrin receptor (TFRC), allowing Fe^3+^ to be released into the cell. In contrast, lactotransferrin can directly shift iron into the cytoplasm (65). TFRC is an important component for iron uptake in the membrane, which can govern the labile iron pool (LIP) by conveying Fe^3+^ into the cytoplasm to promote various biological activities. Fe^3+^ is reduced into Fe^2+^ by STEAP3 once it enters the cytoplasm, and Fe^2+^ is stored in the LIP. Fe^2+^ is important for metabolic and biochemical processes, such as energy metabolism in the mitochondria. TFRC actions alter intracellular iron content, Fe^2+^ levels and reactive oxygen species (ROS) levels. Ye et al. found that (66) TFRC rescued the reduction in iron, Fe^2+^ and ROS concentrations caused by YTHDF1 knockdown in hypopharyngeal squamous cell carcinoma (HPSCC) cells. The study also showed that (67) TFRC might also intervene in glutathione peroxidase 4 (GPX4)-dependent ferroptosis. GPX4 is a key molecule that modulates ferroptosis. In 2021, Ma et al. found that (68) lncRNA RP1-86C11.7 could interact with hsa-miR-144-3p to increase the expression level of TFRC. RP1-86C11.7 enhanced proliferation, migration and progression in glioma. Consequently, accumulation of unstable LIP leads to the overproduction of lipid peroxidation, which is another vital process of ferroptosis in addition to iron metabolism.

### Lipid peroxidation

Lipid peroxidation is a characteristic of ferroptosis that is driven by free radicals, including ROS and reactive nitron species (RNS) (69). During lipid peroxidation, oxidants attack lipids, such as polyunsaturated fatty acids (PUFAs), to produce lipid hydroperoxides (LOOHs) and reactive aldehydes that rely on the catalysis of the ALOX family (70). ROS consist of superoxide anion (O_2_·-), hydroxyl radicals (HO·), hydrogen peroxide (H_2_O_2_) and singlet oxygen (O^2^), which are generated by insufficient reduction of oxygen during hypoxia or in response to other physical and chemical reactions. There are two pathways that produce ROS. One is the NADPH oxidase (NOX) pathway. Another is the Fenton reaction, in which Fe^2+^ interacts with H_2_O_2_ to produce Fe^3+^, HO·and OH-. In return, O_2_·- interacts with Fe^3+^ to produce Fe^2+^. The entire process is called the Haber-Weiss cycle (70). These free radicals contribute to oxidative stress and damage proteins and nucleic acids, a process closely related to the carcinogenic potential in malignant diseases. Bountali et al. demonstrated that (70) lncRNA MIAT knockdown promoted the accumulation of ROS and enhanced cell apoptosis to further influence other cancer-related genes in glioma. It was highly possible that MIAT exerted its effectiveness on ferroptosis by changing ROS levels in glioma. Ahmadov et al. found that (71) N-acetyl cysteine (NAC), a ROS scavenger, could reverse the phenotype caused by the decline of ROS level due to lncRNA HOTAIRM1 knockdown in glioma cells. What’s more, they elucidated that intracellular ROS decrease mediated by HOTAIRM1 contributed to the radiation resistance in glioma. Lulli et al. found that (72) miR-370-3P weakened the proliferation and invasion by directly inhibiting lncRNA NEAT1 in glioma. NEAT1 encouraged the activation of HIF1-α and HMGA1, which were both connected to oxidative stress in glioma. Currently, lncRNA NEAT1 is commonly an oncogene in cancers. Zhen et al. found that (73) lncRNA NEAT1 could be a tumor-enhancer by regulating miR-449b-5p/c-Met axis in glioma. Collectively, these data suggest that the lncRNA NEAT1 may affect ferroptosis by controlling molecules related to oxidative stress in glioma. However, the specific mechanisms by which these lncRNAs affect ferroptosis remain unexplored. In fact, there are many regulators or pathways that modulate intracellular ROS content, such as lipophagy, ferritinophagy, GPX4 and NOXs, and that may be altered by lncRNAs.

### Other potential molecular mechanisms in ferroptosis

The mechanisms of ferroptosis are complicated and obscure. Many molecules, in addition to those mentioned above, are involved in this important biological process (8, 70, 74).. As we all known, glutathione peroxidase 4 (GPX4) is considered as the gatekeeper and hub molecule in ferroptosis. GPX4 belongs to Glutathione peroxidases (GPXs) family, which currently contains GPX1-GPX8. In general, GPXs are involved in the reduction reactions of H_2_O_2_ and small hydroperoxides *via* glutathione (GSH) as reductant. Besides, only GPX4 can catalyze the reduction of hydroperoxides in the complicate lipids, even located in the biomembranes or lipoproteins. Rich evidences (75), have demonstrated GPX4 plays a crucial role in the process of ferroptosis. GSH, comprised of cystenie, glutamate and glycine, is required for GPx4 to execute its functions. Cystine is transported into cells through the protein complex systmeXc- in plasma membrane which consists of SLC7A11/xCT (solute carrier family 7 member 11) and SLC3A2 (solute carrier family 3 member 2). Cystine will be transformed into cysteine to form GSH once it enters into cells. On the other hand, GPX4 can catalyze phospholipid hydroperoxides (PLOOH) to produce phospholipid alcohols (PLOH) and decrease the stock of lipid peroxidants (69, 70). However, to inhibit any one step above will suppress the function of GPX4 directly or indirectly to cause the accumulation of PLOOH to promote ferroptosis. In addition, the stability of SLC7A11 can also influence the occurrence of ferroptosis. Zhao et al. found that (76) OTUB1, an ovarian tumor (OTU) family member deubiquitinase positively regulated the stability of SLC7A11 to support ferroptosis in glioma. Moreover, Liu et al. found that (77) CD44, the biomarker of cancer stem cells directed the process of ferroptosis by promoting the interaction between OUTB1 and SLC7A11, which suggested that CD44 might be involved in the progression of ferroptosis. Chen et al. (78) found that differential expression of lncRNA TMEM161B-AS1 regulated the two ferroptosis-related genes (FANCD2 and CD44) separately by sponging hsa-miR-27a-3p. They also confirmed that depletion of FANCD2 and CD44 caused the accumulation of iron and lipid ROS, suggesting that low expression of lncRNA TMEM161B-AS1 could promote cell apoptosis and ferroptosis in glioma. What’s more, Zhang et al. found that (79) lncRNA OIP5-AS1 inhibited ferroptosis in prostate cancer with long-term cadmium exposure through miR-128-3p/SLC7A11 signaling. Obviously, SLC7A11 played a crucial role in ferroptosis by regulating the transportation of cystine. As mentioned before, oxidative stress involved molecules could cause irreversible or lethal damage to cells. Particularly, NOXs, controlled positively by DPP4/CD26 and other kinases, constitute part of the membrane-bound enzyme complexes that transport electrons necessary for the production of free radicals, including ROS, that promote lipid peroxidation in ferroptosis (69). Another study also reported that (70) DPP4 was involved in the reduction reaction of O_2_ to O_2_·- in a NOXs-dependent manner.

**Figure 2:**
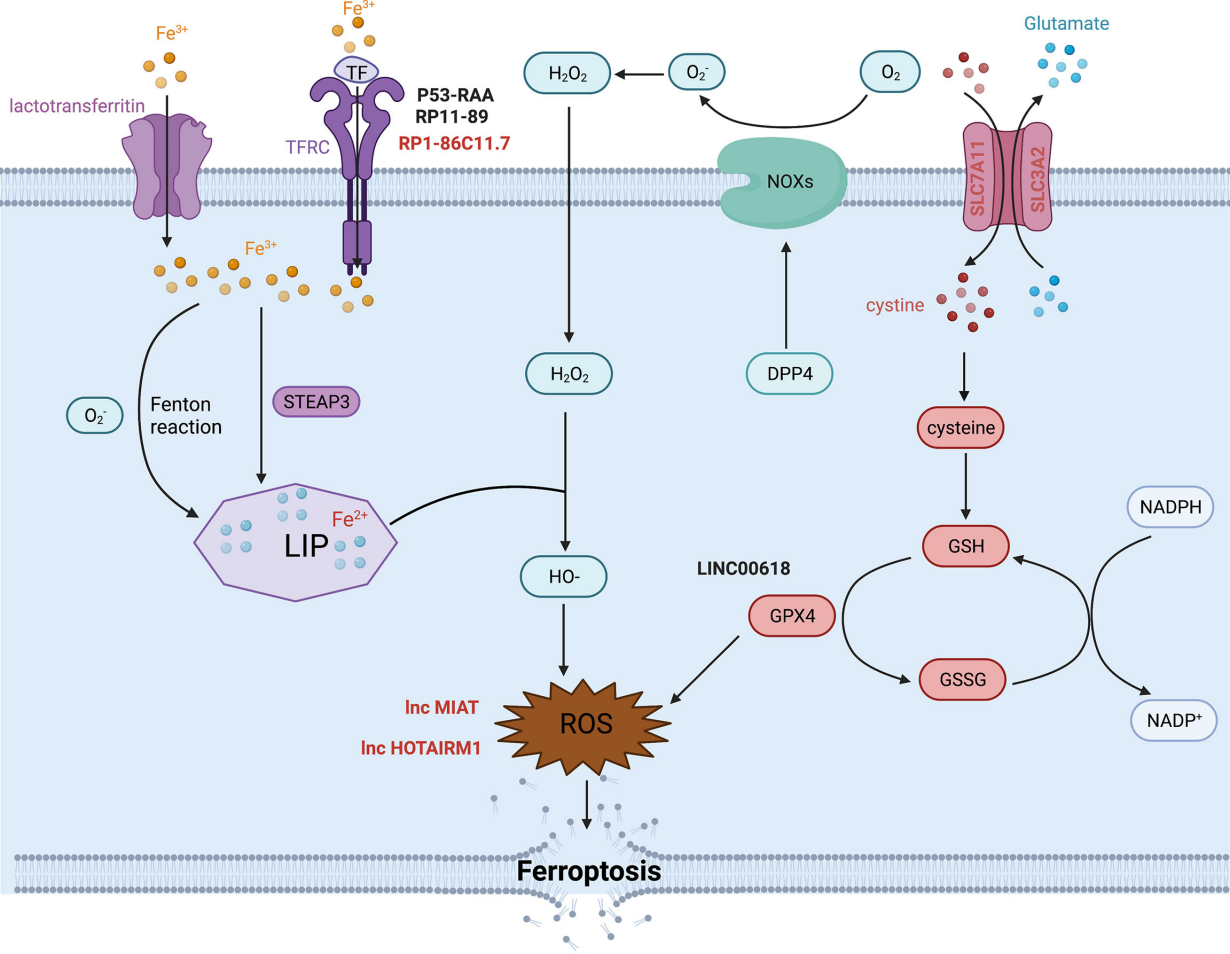
The molecular mechanism and some lncRNAs regulation of ferroptosis.

It is clear that numerous molecules are involved in the complex process of ferroptosis, yet only a few molecules have been declared to influence ferroptosis by interacting with lncRNAs in glioma (**Figure 2**). Ferroptosis is a newly defined process of cell death that plays an important role in the progression of many diseases, especially tumors. Therefore, it is worth exploring the specific and profound mechanisms whereby lncRNAs contribute to glioma to provide new potential targets for therapy.

## Conclusion and prospectives

Glioma is the most prevalent and dangerous CNS tumor with a very poor prognosis. It is noteworthy that GBM patients still remain hopeless prognosis even though they were performed complex treatment scheme combining operation with radio- and chemo- therapy. More depressingly, chemotherapy drugs can’t be easily targeted to GBM due to special location comparing to other non-intra tumors. Therefore, to find some specific targeted molecules for GBM is of vital and urgent. To clarify the pathological molecular mechanisms of GBM is of vital and helps other scientists further explore corresponding target therapy to some extent. And we noticed noncoding RNAs, including miRNAs, circRNAs and lncRNAs play critical and significant roles in suppressing or provoking the initiation and progression of glioma. In particular, lncRNAs which is under the spotlight, impact various aspects of glioma, such as proliferation, invasion, migration, EMT, cell death, stemness of glioma stem cells and resistance to radiotherapy and chemotherapy, by interacting with proteins, mRNAs, enzymes and other noncoding RNAs and interfering countless signal pathways. Thus, it can be seen lncRNAs indeed involves in the development and progression of glioma. However, the specific and precise mechanisms of lncRNAs still need to be further probed in future. For example, whether lncRNAs involves in the ferroptosis of glioma or not? And can lncRNAs put an effect on the ferroptosis progress by some molecules or similar pathways involved in the proliferation, EMT, apoptosis and TMZ of glioma?

Recently, ferroptosis, a novel class of cell death, has attracted much attention in various diseases. However, few articles have discussed the mutual interaction between lncRNAs and ferroptosis in glioma compared with other diseases. Similar relationships between lncRNAs and ferroptosis found in other diseases may also be present in glioma. For instance, Mao et al. (80) reported that lncRNA P53RAA promotes ferroptosis by accumulating iron and lipid ROS and displacing p53 from the G3BP1-p53 complex. Simultaneously, P53RAA can decrease the expression of the metabolic molecule SLC7A11, a regulator of iron concentration. It is well-known that p53 is a classical tumor suppressor. Therefore, the following question arises: can p53-related lncRNA induce ferroptosis in glioma (81)? Besides, He et al. (82) and Shi et al. (83) built a novel ferroptosis-related lncRNAs panel which provides some assertive evidence for delving into the relationship between lncRNAs and ferroptosis in glioma. Meantime, they released some implications and values for the potential therapy plan related to immunotherapy for glioma patients.

Glioma is different from other tumors due to its heterogenity and has a special tumor microenvironment (TME) consisted of cancer cells and immune cells, including macrophages, nature killing cells, dendritic cells and et al. The growth of glioma cells is dependent on the iron element comparing with other non-malignant cells (84). Moreover, glioma mostly occurred in the brain and iron is usually transported mediated by TFR into the brain, which indicated that we can induce the ferroptosis to consume the iron to prevent the growth of malignant cells. However, how to achieve it? As mentioned above, lncRNAs can act various role in the progress of ferroptosis in glioma. If lncRNA X (which means any suitable lncRNA), high tissue-specificity, were found to induce ferroptosis by increasing the iron concentration or TFR in brain, we can focus on to synthesize a kind of drug to change the expression of lncRNA X to induce ferroptosis to obstruct the proliferation of glioma cells. Besides, we can monitor the change of lncRNA X level in tissue to hint us to take precautions against the glioma. Furthermore, human brain, enriched in lipid, is the most susceptible to the progress of oxidative stress reaction which also are very meaningful to ferroptosis (85). It is possible for others to develop some related and helpful therapy plans targeting lncRNAs involved in ferroptosis followed the same mind above accordingly. Several works have showed that the links between immune cells within TME and ferroptosis implicated that some immune therapy targeting ferroptosis might can be explored (86, 87). More promisingly, it has been verified that CD4, CD8 and CD36 T cell within TME can induce the ferroptosis by accumulating the lipid ROS (88), which suggest we can mainly concentrate on some immune-related lncRNAs involved in the ferroptosis to scout the links and the potential therapy. In addition, the induction of ferroptosis can prevent the formation of acquired drug-resistance which is significant and meaningful clinically.

Ferroptosis is a complex process and a newly discovered modality of cell death. The interactions between ferroptosis and other processes of cell death have been explored, as the mechanisms of ferroptosis have become increasingly clearer. Wang et al. (89) found that LINC00618 knockdown reduced early apoptosis. In addition, LINC00618 can inhibit GPX4, a key regulator of ferroptosis, and increase the concentration of intracellular iron and lipid ROS. Ultimately, they suggested that LINC00618 can increase ferroptosis in a manner dependent on cell apoptosis. Therefore, we suggest that lncRNAs might act as bridge molecules between ferroptosis and

apoptosis, including cellular and necrotic apoptosis. Moreover, this evidence highlights the potential crosstalk or interrelationship amongst cell apoptosis, necrosis, autophagy and ferroptosis that may occur in or to contribute to many diseases and should be the focus of future studies.

In conclusion, lncRNAs play a crucial role in the occurrence and development of glioma. Targeting these lncRNAs may help glioma patients to obtain potential treatment benefits. In addition, the identifications of lncRNAs may contribute to the early detection and diagnosis of glioma. However, in order to fully understand the function of lncRNAs in the neoplastic process of glioma, several key issues must be solved. For example, since lncRNAs have various functions and can regulate a variety of cellular processes, it is necessary to analyze the specific molecular mechanisms of it. In addition, whether the participation of lncRNAs in clinical application has sufficient reliability and sensitivity or not remains to be verified. We believe that the use of robust sequencing techniques can shed light on the roles of lncRNAs in glioma development and could accelerate the clinical application of lncRNAs in diagnosis, treatment, and prognostic evaluation of glioma.

## Author Contributions

TX, QL, XY, and JG conceived the concept and wrote the manuscript. ZL, MH, XJ, ZW, YL, DH, and FZ finished the table and figure. All authors read and approved the final manuscript.

## Funding

This work was supported by Natural Science Foundation of China (Grant NO. 81972340, 82173140,81871196), Science and Technology Project of Jinan city (Grant NO.201907048), Shandong Provincial Natural Science Foundation, China (GrantNo.ZR202010300086), Key Projects of Natural Science Foundation of Jiangxi Province (Grant NO. 20192ACB20011), Academic promotion program of Shandong First Medical University (Grant NO. 2019LJ005).

## Conflict of Interest

The authors declare that the research was conducted in the absence of any commercial or financial relationships that could be construed as a potential conflict of interest.

## Publisher’s Note

All claims expressed in this article are solely those of the authors and do not necessarily represent those of their affiliated organizations, or those of the publisher, the editors and the reviewers. Any product that may be evaluated in this article, or claim that may be made by its manufacturer, is not guaranteed or endorsed by the publisher.
